# Is there any Influence of Variations in Context on Object-Affordance Effects in Schizophrenia? Perception of Property and Goals of Action

**DOI:** 10.3389/fpsyg.2016.01551

**Published:** 2016-10-05

**Authors:** Jessica Sevos, Anne Grosselin, Denis Brouillet, Jacques Pellet, Catherine Massoubre

**Affiliations:** ^1^Department of Psychiatry, University Hospital of Saint-ÉtienneSaint-Étienne, France; ^2^TAPE Laboratory, EA7423, University of Jean MonnetSaint-Étienne, France; ^3^Epsylon Laboratory, EA4556, Department of Psychology, University of Montpellier IIIMontpellier, France

**Keywords:** context, embodiment, goals of action, object-affordance effect, schizophrenia, sense of property, sensorimotor simulation, Stimulus–Response-Compatibility

## Abstract

The simple perception of an object can potentiate an associated action. This affordance effect depends heavily on the action context in which the object is presented. In recent years, psychologists, psychiatrists, and phenomenologists have agreed that subjects with schizophrenia may not perceive the affordances of people or objects that could lead to a loss of ease in their actions. We examined whether the addition of contextually congruent elements, during the perception of everyday objects, could promote the emergence of object-affordance effects in subjects with schizophrenia and controls. Participants performed two Stimulus–Response-Compatibility tasks in which they were presented with semantic primes related to sense of property (Experiment 1) or goal of action (Experiment 2) prior to viewing each graspable object. Controls responded faster when their response hand and the graspable part of the object were compatibly oriented, but only when the context was congruent with the individual’s needs and goals. When the context operated as a constraint, the affordance-effect was disrupted. These results support the understanding that object-affordance is flexible and not just intrinsic to an object. However, the absence of this object-affordance effect in subjects with schizophrenia suggests the possible impairment of their ability to experience the internal simulation of motor action potentialities. In such case, all activities of daily life would require the involvement of higher cognitive processes rather than lower level sensorimotor processes. The study of schizophrenia requires the consideration of concepts and methods that arise from the theories of embodied and situated cognition.

## Introduction

Embodied theories of cognition address the physical, motivational, and environmental dimensions of an individual’s daily experience (Varela et al., 1992). Such a view posits that cognition emerges from the cooperation and co-evolution of perceptual and motor systems that allow sensorimotor patterns to be implemented. Perception is therefore more proactive toward than reactive to the individual’s environment (Berthoz and Petit, 2003; Rizzolatti and Matelli, 2003; Gallese, 2007; Barsalou, 2008, 2009; Vandevoorde, 2011); it’s an internal simulation of action, designed for understanding the meaning of his environment (Berthoz, 2003). The subject builds the world in which he lives and acts, in accordance with his perception of that environment (Glenberg et al., 2013) and the actions he performs within it. Consequently, he perceives through active exploration and intentional activity according to his goals, tempered by intrinsic constraints of perceptual systems and context, and not simply by interpreting sensory messages (Haggard, 2005). This binding of action and perception allows the most economical solution to emerge from a set of possible actions. Gibson (1977, 1979) described “affordance” as the subject’s faculty to guide his or her behavior, according to the perception of what the environment is offering in terms of action potentialities.

In cognitive psychology, numerous studies have operationalized this concept of affordance, (e.g., Tucker and Ellis, 1998, 2001, 2004; Ellis and Tucker, 2000; Borghi et al., 2012). In their seminal study, Tucker and Ellis (1998) associated the orientation of the typical graspable part of an object with the hand used by a participant to respond to instructions regarding the object, observing that this compatibility of orientations facilitates response. They showed that perception of an object automatically potentiates related actions (via simulation mechanisms) even in the absence of instruction or explicit intention to act.

However, others describe an implicit motor intentionality to act on an object as a basis of the affordance effect. Naming an object automatically elicits action potentialities not elicited by its passive viewing alone; its naming evokes gestural knowledge of the object’s form and function (Bub and Masson, 2006). The behavioral impact of perceived object affordance seems to depend heavily on the action context in which the object is presented to carry on the subject motor intentionality.

These action potentialities could match behavior or attitude sketches (“covert behaviors”) that the individual reenacts as he perceives environmental stimuli, and these simulations of action (Berthoz and Petit, 2006) might represent a third component that requires incorporation into the relation between perception and action (Garbarini and Adenzato, 2004). At the neuronal level, the brain employs similar neural resources and dynamic representations to execute, imagine, and perceive actions (Jeannerod, 2006; Barsalou, 2008). This ability to simulate an action in the absence of its effective implementation, which is underpinned by “canonical” and “mirror” neural networks, give meaning to the surrounding world (subjects and objects). In this case, knowledge is enacted and carries the implicit meaning of perceived world.

### Disembodiment in Schizophrenia

“This tacit or enacted knowledge is also the basis of “common sense” (Blankenburg, 2001; Fuchs, 2001): it provides a fluid, automatic, and context-sensitive pre-understanding of everyday situations, thus connecting self and world through a basic habituality and familiarity” (Fuchs, 2015, p. 199).

Embodied theories of cognition highlight the dependence of cognition on the subject’s experience of the world without addressing mental pathologies, especially schizophrenia.

However, some approaches to psychopathology that consider the phenomenological dimension of embodiment (Sass and Parnas, 2003; Fuchs, 2005) describe schizophrenia as a disturbance of the individual’s relationship with the world and others that constitutes a “lack of common sense” (Stanghellini, 2000).

From a clinical perspective, therapists report that their patients with schizophrenia experience some perceptual or cognitive fragmentation of the world accompanied by a certain loss of ease in their actions. As a result, they may experience a disintegration of habits or automatic practices. Due to an alteration of the body-based involvement, patients have to “think” deliberately about each action, before to perform it. Sass (2004, p. 136) describes the failure of patients with schizophrenia to perceive the affordances of people, things, or actions that give the objects “*practical significance that, for example, make a chair a thing-to-sit-on, a hammer something-to-pound-with, or a human body something to be approached, feared, or caressed*.”

Until now, experimental studies have focused on the impairment in the motor understanding of other’s behaviors in schizophrenia, as revealed by a reduced activation of the mirror neural network. Numerous studies in patients show an inherent deficit that inactivates this neural network (Mehta et al., 2014a). Using different paradigms, most studies showed reduced mirror neuron activity (MNA) and greater deficits in theory of mind (Mehta et al., 2014b), emotion recognition (Mehta et al., 2014b), and expression (Varcin et al., 2010), action imitation (Park et al., 2008; Thakkar et al., 2014), and observation (Enticott et al., 2008) in patients. If this mirror neural network enables the internal simulation of behaviors, it is not surprising that individuals with schizophrenia have difficulties relating with the world in interpersonal relationships (Mehta et al., 2014b).

However, to our knowledge, only few studies extend this line of research to object’s perception. For example, Delerue and Boucart (2012) noted the absence of a perception-action link in schizophrenia when they measured eye-tracking during an active visual scanning task to show the decoupling of an object’s perception from the potential action. They demonstrated that the visual exploration of control subjects varied according to the instructions given, to name the object visually presented or to name the action inferred by the object. When participants had to name the object, they explored only the part useful for its identification, focusing, for example, on the tines of a fork. However, when they had to name the action, they explored the whole object, extending their visual explorations, in this case, to the handle, the graspable part of the fork. In contrast, visual explorations of patients were similar for the two tasks; in each case, they focused essentially on the useful part (tines) to identify the object (fork). Though patients with schizophrenia demonstrated no abnormalities in naming objects, the absence of facilitation in naming the actions of objects could reflect impaired perception of affordance.

Our team also studied the affordance in schizophrenia (Sevos et al., 2013). Since some authors suggested that “[i]*f sensory and motor processes are basic to all other cognition, as much research in embodied cognitive science posits, then disorders that have traditionally been viewed as dysfunctions of higher cognitive processes could in fact be explained by lower level sensorimotor processes.*” (Drayson, 2009, p. 338), we proposed to study a potential deficit of sensorimotor integration, instead of higher cognitive dysfunction, in this pathology. We evaluated whether perceived objects automatically evoke corresponding action processes (sensorimotor integration) using a Stimulus–Response-Compatibility (SRC) paradigm that provides for a shorter response time when the stimulus and response share the same properties (Sevos et al., 2013). In our first experiment (Experiment 1), we observed faster response times when the spatial localization of a stimulus and of the motor response were compatible (Simon effect), and patients with schizophrenia showed no impairment of visuo-spatial integration in this task. In our second experiment (Experiment 2), we replicated the tasks of Tucker and Ellis (1998) that measured the effect of compatibility between the orientations of common graspable objects and the hand with which the subject was to respond to instructions (object-based affordance effect). The absence of this effect even in patients with mild symptom severity suggested no automatic binding of perception and action in this population.

If a relationship between the features of a motoric object and the action to be carried out with it does not occur automatically, is it possible that adding contextual elements, making the action more relevant to the patient’s needs and wishes, could induce this automatic link between perception and action?

Indeed, in controls, it is known that the context in which an object is observed influences how the object is perceived. The activation of action potentialities, such as grasp, is not completely automatic but depends rather on how attention is oriented toward the action-relevant features of an object. For example, the perception of the same object (door handle) could trigger different sensorimotor simulations (Tipper et al., 2006). Affordance effects were obtained only when subjects had to discriminate properties of an object linked to action (a shape); they did not occur when they had to discriminate color.

### The Influence of Variations in Context on Object-Affordance Effects

An individual’s range of potentially available motor actions also depends on his unique “history” of interactions between the object perceived and actions carried out with it. Moreover, the context acts as either a resource or constraint according to the situation, the subject’s ability to exploit environmental resources, and the subject’s goals of action (Creem and Proffitt, 2001). Therefore, it is not surprising that there is widespread interest in the modulation of affordance effects in various experimental contexts (Borghi et al., 2012; Borghi and Riggio, 2015). Researchers disagree regarding the degree of automaticity of action potentiations versus task- and context-dependent activations (Creem-Regehr and Lee, 2005; Buxbaum and Kalenine, 2010; Borghi and Riggio, 2015). Nevertheless, efforts have been made to clarify these questions by modifying the context of experimental settings. Among such studies, we will emphasize those that address goals of action and sense of property.

### Goals of Action

The context in which an object is observed has been shown to influence how it is perceived. Some authors believe that affordance effects are not immutable but may vary according to the observer’s goals and intentions in a given environment: the perception of one object might trigger different sensorimotor simulations. The simultaneous automaticity of the activation of affordance and the flexibility of its modulation according to the task and the physical and social context has been recently shown (Borghi and Riggio, 2015) as well as the same object can evoke different affordances (manipulative or functional grip) according to context (Kalénine et al., 2014). In the same idea, some authors have demonstrated a motor facilitation when two objects are congruent and disposed in a functional way to imply a specific action (Yoon et al., 2010), and that presenting a photo of a hand with prehensile posture congruent with that of the hand with which the subject was to respond facilitated the response to objects (Borghi et al., 2012). By implying the individual’s underlying goal, the photo would more strongly induce interaction between the object and action. A facilitation effect in conditions of both functional congruency between two objects (presenting together a bottle and glass rather than a bottle and ball) and the status of the objects (presenting together a bottle and empty glass rather than a bottle and full glass) was also reported (De Stefani et al., 2012). The object’s state also appears to influence affordances, since larger effects were observed when the perceived object appeared active (a door handle that was depressed) rather than passive (the door handle in apparent inactivity) (Tipper et al., 2006). All these studies showed the flexible experience of objects by individuals and the modulation of their perception according to their use in a given context.

Semantic material has also been used to investigate the modulation of affordance effects according to goals of action. Indeed, language, as memory or perception, implies sensorimotor simulation mechanisms linked to objects or situations to which these linguistic expressions refer (e.g., Glenberg and Kaschak, 2002; Zwaan and Taylor, 2006; Gallese, 2009; Borghi and Pecher, 2011). In particular, words evoke object affordance just as visual stimuli do (Gibson, 1979). This functional link between language and motor systems results from the often simultaneous occurrence of actions and their referents; neural populations, recruited to process a word and the referent body movement, frequently fire together and become strongly linked (Pulvermüller, 1999, 2001).

Borghi and Riggio (2009) showed that reading a sentence as a prime of an visual object automatically activate the goal of the action but only when the sentence included a verb of action compared with a verb of observation. They also observed an interference effect when the action sentence and perceived object were incongruent. Using a similar design, Costantini et al. (2011) observed the triggering of a simulation effect by an action sentence, but only when the proximity of objects permitted bodily interaction (i.e., in the peripersonal space). Thus, specifying the proper conventional use of an object encourages the simulation of a particular pattern of motor response.

On the other hand, some authors do not show that semantic context leads to an automatic and invariant simulation of specific motor programs (Van Dam et al., 2010). Indeed, in a functional magnetic resonance imaging task that involved words with both motor and visual characteristics, such as tennis ball and boxing gloves, stronger activation of motor areas when subjects thought about words with motor characteristics was reported (Van Dam et al., 2012). These findings suggest that the activation of motor-specific information during action-word comprehension is flexible and contextually dependent.

### Sense of Property

Affordance effects can depend on the actor’s sense of who owns the objects with which he interacts. This sense of property as consider as a basic mechanism that emerges automatically even during tasks not directly related to ownership (Tummolini et al., 2013). Using a SRC paradigm, Constable et al. (2011) showed variation in action potentialities evoked during the perception of objects according to the individual’s understanding of who owned the object. They asked participants to decorate a cup and use it at home to create the feeling that they owned the cup. Fifteen days later, participants performed a task on computer that used photos of different cups: their own, one decorated by the investigator, and two others with no defined owner. Sensorimotor compatibility effects were shown for all photos except those of the investigator’s cup. The authors conclude that a sense of ownership may be embodied in the visuomotor system that is sensitive to the status of an object’s ownership and favors the inhibition of action that involves another person’s objects rather than the facilitation of action toward self-owned objects. These data fit with the hypothesis of an early developmental sense of property. Indeed, as early as age four, children understand that inappropriate interaction with objects they do not own can result in negative consequences (Neary et al., 2009).

The sense of property is partly determined by one’s identity (Dittmar, 1992), and name and surname are essential components of that identity. The repetition of an individual’s names throughout daily life could automatically draw auditory and visual attention to these words (Moray, 1959; Wood and Cowan, 1995; Shapiro et al., 1997) and evoke more memories and emotions than other words do. The detection of one’s surname among other stimuli has been demonstrated on both behavioral (Oswald et al., 1960) and cerebral levels (Perrin et al., 1999), even during sleep. One’s surname seems to be a pertinent ecological stimulus for reference to “self” (Perrin et al., 2005). Markman and Brendl (2005) presented individuals’ own surnames in the middle of a screen and asked participants to categorize the valence of positive or negative adjectives placed near or far from their surname. Half of the participants were instructed to pull a lever toward themselves in response to positive adjectives and to push it away in response to negative adjectives, and the other half were given the reverse instructions. Participants responded faster when positive adjectives were closer to their surname and when negative words were further away, irrespective of the participant’s pushing or pulling the lever. The authors concluded that the speed of response movements depended more on the representation of the participant’s self-the subject’s surname on the screen-than the representation of their body, the physical activity of pushing or pulling of the lever.

If attentional processes can automatically be attracted by self-relevant items (as participant’s names -Moray, 1959; Gray et al., 2004- or object ownership -Turk et al., 2011), we can then expect that this kind of stimuli have an impact on other cognitive or behavioral processes.

### The Present Study

We examined whether the addition of a more salient action context can promote the emergence of affordance effect during the perception of everyday objects in patients with schizophrenia. Indeed, in this population, the simple perception of an object without context does not automatically evoke corresponding action processes, and this lack of sensorimotor integration (Sevos et al., 2013) could be associated with the absence of visual exploration of the action-relevant features of the object (Delerue and Boucart, 2012).

In this study, we explored the emergence of object-affordance effects in schizophrenia using variations of new experimental contexts in two experiments focusing on action potentiation during the perception of object handling. In the first experiment (Experiment 1), we began with the SRC paradigm of Tucker and Ellis (1998) and added the presentation of primes (surnames) to enhance the context, using the participant’s surname or “Rani” as an imaginary surname to act as a reference of owning or not owning the perceived object. In the second experiment (Experiment 2), we added action sentences primes that were congruent or not with the goal of action induced by the conventional use of a given object. For example, we would present the sentence “For watering plants” followed by a photo of a watering can or a remote control.

## Experiment 1

To evaluate if the sense of property can modulate object’s affordance effects (in controls and patients with schizophrenia), we introduced surnames, known to be ecological stimuli for self-reference (Markman and Brendl, 2005; Perrin et al., 2005), as primes to enhance the context of an object.

Because people are also known to interact with objects differently based on whether the objects belong to them or not (Constable et al., 2011), we considered the affordance effects related to the introduction of surnames to reference the subject’s owning (participant’s name) or not owning (imaginary name, “Rani”) the perceived objects. We expected the emergence of affordance effects in both populations when objects were primed with the participant’s surname but not the imaginary name.

### Materials and Methods

#### Participants

Participants included 18 patients with schizophrenia (16 men, 2 women) recruited in the psychiatric departments of the University Hospital of Saint-Étienne and 18 healthy comparison subjects (15 men, 3 women) recruited by advertisement in the local newspaper (See **Table 1** for demographic comparisons). All were volunteers and naive about the hypothesis of the experiment. The local ethics committee of Saint-Étienne approved the study (N° IORG0007394), and written consent was obtained from all participants after the nature of the procedures was fully explained.

**Table 1:** Comparison of ages, years of education, and scores on the Edinburgh Handedness Inventory (SD) between patients with schizophrenia and control subjects in Experiment 1.

	Patients (*n* = 18)	Controls (*n* = 18)	*t*-test; *P*-value
Age (years)	37.3 (6.9)	36.9 (8.8)	*t* = 0.126; *P* = 0.900
Education (years)	12.1 (2.5)	11.2 (2.7)	*t* = 0.949; *P* = 0.349
Edinburgh score (/20)	19.3 (1.1)	18.6 (1.7)	*t* = 1.552; *P* = 0.130
PANSS positive	13.1 (3)	/	/
PANSS negative	16.7 (7)	/	/
Illness duration (months)	172.33 (93.9)	/	/

Patients were included with a DSM-5 diagnosis of schizophrenia (American Psychiatric Association, 2013) and no change in antipsychotic medication and/or clinical status within 4 weeks prior to the study. The same senior psychiatrist assessed all patients using the positive and negative syndrome scale (PANSS; Kay et al., 1987). All were stable outpatients living in their own accommodations and participating in various psychosocial or professional activities.

Across groups, participants were excluded for (1) a diagnosis of neurological brain disorder or head trauma with loss of consciousness, (2) mental retardation, and/or (3) a history of substance abuse over the last 6 months. All participants were right handed (scores > 14, assessed using the modified Edinburgh Handedness Inventory; Oldfield, 1971).

A power analysis, conducted via G^∗^Power Software (Faul et al., 2007), with Cohen’s recommendations (Cohen, 1988), which assumed a medium effect size of 0.25 for the ANOVA with one between-subjects factor and three within-subjects factors (eight levels as repeated measures), indicated that a total of 20, 18, and 16 participants were required, respectively, to have a 90, 85, or 80% power (a minimum required by Cohen, 1988) of detecting a significant effect at *p*-value of 0.05. Thus, our proposed sample size of 36 subjects will be more than adequate for the main objective of this study. The power analysis which assumed a medium effect size of 0.25 for the ANOVA with three within-subjects factors in each group of participants (controls and schizophrenics) indicated that a total of 20, 18, and 16 participants were required to have 90, 85, and 80% power of detecting a significant effect at *p*-value of 0.05. In the present study, 18 subjects performed all conditions in each group.

#### Apparatus and Materials

We employed the same material we used previously (Sevos et al., 2013), adding only a visual prime (participant’s surname or the imaginary surname “Rani”) before presenting each object in each trial. We chose an imaginary name to avoid reference to any of the participants. Names printed in black 32-point Arial font were presented to subjects in the middle of a white screen.

**FIGURE 1:**
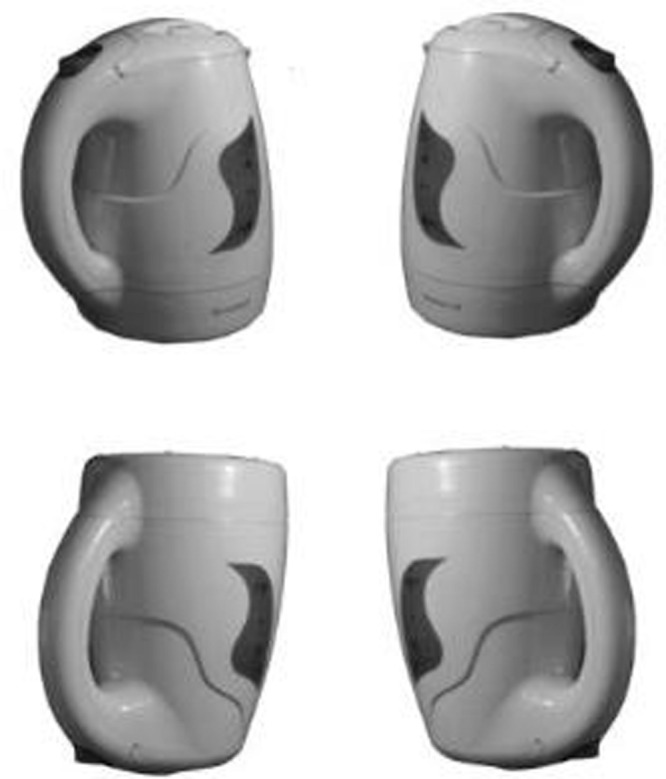
**Examples of the stimuli used in Experiment 1: left orientation, upright; right orientation, upright; left orientation, inverted; right orientation, inverted**.

A total of 88 black-and-white photographs of 22 objects graspable by one hand (Appendix 1) were presented in two horizontal (compatible with either a right- or left-handed grasp) and two vertical (upright or inverted) orientations (**Figure 1**) on the computer screen. The average size of photos was 512 × 384 pixels to maintain the proportions of each object at a distance of 50 cm.

#### Design and Procedure

Participants were seated with their heads 50 cm in front of the screen and first shown every photo in both orientations to ensure they could recognize each object upright and inverted.

During the task, they were required to indicate as quickly as possible whether the object was upright or inverted by pressing the corresponding response key. Each participant carried out two blocks of 88 trials with a break of 3 min between blocks. In one block, subjects were to respond with their right hand for upright objects and their left hand for inverted objects. In the second block, they were asked to do the opposite – to respond with their right hand for inverted objects and their left hand for upright objects. The order of these blocks was counterbalanced between subjects. Within each block, 44 randomized trials were primed by the participant’s surname and the other 44, by the imaginary surname “Rani.” Before carrying out each block, the subject was informed that his surname would appear before objects as if he owned them or the surname Rani would appear before objects as if Rani owned them, and they were reminded to respond to objects according to the object’s orientation and not the presented surname. A 1-min break was proposed after the first 44 trials. The order of primes was also counterbalanced between participants. Moreover, for each participant, objects primed by their own surname were never primed by Rani, and objects primed by Rani were never primed by their own surname, and the order of presentation was counterbalanced between subjects. During the whole experiment, participants were instructed to keep their right finger on a right response key (L) and their left finger on a left response key (S). Response keys were situated 15 cm apart and 20 cm in front of the screen, on a standard European (AZERTY) keyboard.

Each participant received six practice trials for each kind of prime (surname of participant or Rani) before each block. Each experimental trial started with the presentation of the prime in the center of the screen for 250 ms followed immediately by a photo of one of the 22 objects. The stimulus stayed on the screen until an answer was given or up to 3000 ms. A brief auditory tone on the computer indicated errors to participants.

### Results

For all conditions, participants responded within the required time limit of 3000 ms. An analysis of variance (ANOVA) was conducted on the participants’ data (errors and response times [RT]) with group (patients or controls) as the between-subject factor and prime (congruent or incongruent), response (left or right hand) and object orientation (left or right) as the within-subject factors.

#### Errors

Errors were rare (*M* = 3.64%, standard error [SE] = 0.5). The ANOVA showed no main effect of either between- or within-subject factors: group [*F*_(1,34)_ = 3.300; *P* = 0.078; η^2^ = 0.09]; prime [*F*_(1,34)_ = 0.027; *P* = 0.871; η^2^ < 0.01]; response [*F*_(1,34)_= 0.156; *P* = 0.695; η^2^ < 0.01]; and orientation [*F*_(1,34)_= 0.041; *P* = 0.840; η^2^ < 0.01] (**Table 2**). Neither did we find interaction among factors (all *F*-values were less than 2.152).

**Table 2:** Error rates (SD) based on patient or control group, congruent or incongruent prime, left or right orientation, and left- or right-handed response in Experiment 1.

		Patients (*n* = 18)		Controls (*n* = 18)	
Response hand	Object orientation	Primes			
		Congruent	Incongruent	Congruent	Incongruent
Right	Right	3.6 (1.4)	5.6 (1.5)	2.0 (0.8)	2.6 (0.6)
	Left	5.6 (1.4)	4.7 (1.1)	1.4 (0.6)	3.1 (1.3)
Left	Right	4.7 (1.2)	5.3 (2.0)	2.6 (1.0)	2.4 (0.8)
	Left	4.7 (1.6)	3.6 (1.3)	4.1 (0.9)	2.3 (0.6)

#### Response Times

The mean RT and standard deviation (SD) were calculated for each subject, and response times above 2 SDs of their own individual mean were eliminated (4.3%).

We found no effect of vertical orientation [*F*_(1,34)_= 0.926; *P* = 0.342; η^2^< 0.03] or mapping responses [*F*_(1,34)_= 1.985; *P* = 0.168; η^2^ < 0.06].

Globally, an increased RT of patients (*M* = 783 ms; *SE* = 24) compared with controls (*M* = 671 ms; *SE* = 23) was reflected by a significant main effect of group [*F*_(1,34)_= 11.492; *P* = 0.002; η^2^= 0.25]. We found no main effect of prime [*F*_(1,34)_= 0.134; *P* = 0.717; η^2^< 0.01]. RTs did not differ significantly when the object was primed by either the participant’s surname (*M* = 724 ms; *SE* = 19) or the surname Rani (*M* = 729 ms; *SE* = 17). Neither did we find an effect of response [*F*_(1,34)_= 0.233, *P* = 0.633; η^2^< 0.01] or orientation [*F*_(1,34)_= 0.007; *P* = 0.935; η^2^< 0.01]. The only significant interaction was between group, prime, response, and orientation [*F*_(1,34)_= 8.536; *P* = 0.006; η^2^= 0.21]. To facilitate reading, we will present separately the analysis according to type of prime (incongruent or congruent) and according to group (patients and controls) (**Table 3**).

**Table 3:** Means (SD) of response times (in ms) according to patient or control group, congruent or incongruent prime, left or right orientation, and left- or right-handed response in Experiment 1.

		Patients		Controls	
Response hand	Object orientation	Primes			
		Congruent	Incongruent	Congruent	Incongruent
Right	Right	786 (32)	774 (22)	658 (23)	679 (26)
	Left	785 (28)	790 (27)	677 (24)	674 (23)
Left	Right	776 (27)	789 (27)	678 (31)	676 (24)
	Left	787 (35)	777 (23)	647 (24)	677 (22)

In the incongruent prime condition, group [*F*_(1,34)=_ 2.638; *P* = 0.114; η^2^ = 0.07] did not change the compatibility effect (measured by the interaction of response × orientation), but in the congruent prime condition, the 3-way interaction of group × response × orientation was significant [*F*_(1,34)=_ 6.202; *P* = 0.018; η^2^ = 0.15]. When the prime was the participant’s name, the temporal patterns of responses, which highlighted the effects of compatibility and incompatibility, differed significantly according to the group of participants.

**FIGURE 2:**
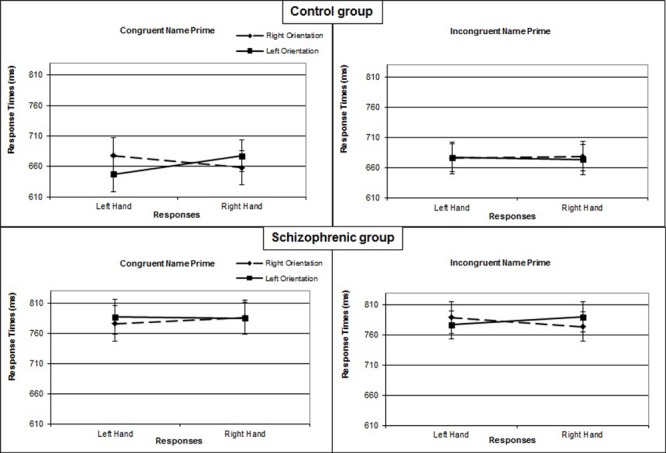
**Mean response times (in ms) for Experiment 1 as a function of name prime (congruent or incongruent), object orientation (left or right), and response hand (left or right) in the control group and in the group with schizophrenia**. Error bars represent standard errors of the mean.

In the control group, the interaction of response × orientation, which measures the compatibility effect, was significant [*F*_(1,17)_= 5.255; *P* = 0.035; η^2^= 0.24], as was the 3-way interaction of prime × response × orientation [*F*_(1,17)_= 6.642; *P* = 0.020; η^2^= 0.28]. For this group, in the congruent prime condition, right-hand responses were faster when the orientation of the object was also to the right (*M* = 658 ms; *SE* = 23) rather than left (*M* = 677 ms; *SE* = 24) [*F*_(1,17)_= 6.582; *P* = 0.020; η^2^= 0.28]. Similarly, left-hand responses were faster when the orientation of the object was also to the left (*M* = 647 ms; *SE* = 24) rather than the right (*M* = 678 ms; *SE* = 31) [*F*_(1,17)_= 5.802; *P* = 0.028; η^2^= 0.25]. The interaction of response × orientation was significant [*F*_(1,17)_= 9.602; *P* = 0.007; η^2^= 0.36]. By contrast, in the incongruent prime condition, the interaction of response × orientation was not significant [*F*_(1,17)_= 0.358; *P* = 0.558; η^2^= 0.02] (**Figure 2**).

In the patient group, the interaction of response × orientation was not significant overall [*F*_(1,17)_= 0.354; *P* = 0.560; η^2^= 0.02], not modified by the prime condition [*F*_(1,17)_= 2.584; *P* = 0.126; η^2^= 0.13], nor significant in either the congruent [*F*_(1,17)_= 0.390; *P* = 0.541; η^2^= 0.13] or incongruent prime condition [*F*_(1,17)_= 2.458; *P* = 0.135; η^2^= 0.13] (**Figure 2**).

### Discussion of Experiment 1

Response times of controls were shorter when the graspable part of the object and the response hand were compatible but only when the subject’s surname was used to prime the object. When an imaginary surname was used as the prime, no compatibility effect was apparent, findings in accord with those of Constable et al. (2011) that specifying the owner of the perceived object modulated affordance effects. Participants might view their own surname as a request to perform a certain action with the object, whereas seeing another name might be interpreted that the other person should perform the action. Therefore, using a participant’s own name as a prime seems to be an ecological way to lead subjects to build a sense of property of the perceived object.

Nevertheless, this kind of prime seems insufficient to create the sense of property in schizophrenia. Patients did not respond faster in the case of compatibility than that of incompatibility whether the objects were primed using their own or an imaginary name. In this group, even when primed by the subject’s surname, the visual perception of an object did not potentiate the actions “normally” associated with it. So, we can assume that for patients with schizophrenia, this type of prime is not sufficiently relevant to allow the emergence of sensorimotor compatibility between an object and action to perform.

Globally, responses of subjects with schizophrenia were slower even if they committed no more errors than controls and whether or not the name used as a prime was their own. Thus, it seems that additional cognitive cost is required of patients to achieve the same results. More costly in attentional resources, the implementation of controlled processes is required if the motor simulation does not emerge when the stimulus and response share sensorimotor characteristics.

The use of the participant’s first name as a prime seems insufficient for patients to perceive the action-relevant features of prehensile objects. Indeed, the behavioral impact of perceived object affordance seems to depend heavily on the action context in which the object is presented, carrying on the subject motor intentionalities. Studies in healthy subjects have shown, for example, that naming an object evokes gestural knowledge about its form and function that automatically elicits action potentialities, whereas passively viewing the object does not (Bub and Masson, 2006).

In a second experiment, to facilitate the perception of the action-relevant features of an object and carry on an implicit motor intention in the patient, we primed objects using action sentences reflecting congruency with the use of the objects in everyday life.

## Experiment 2

If context and goal of action can modulate affordance effects (Borghi et al., 2012), the use of action sentence primes with sensorimotor characteristics congruent with the goal of action induced by the conventional use of a presented object should produce affordance effects in both groups. However, this effect should not emerge when objects are primed using incongruent sentences.

### Materials and Methods

#### Participants

Subjects were 18 patients with schizophrenia (15 men, 3 women) and 18 healthy controls (15 men, 3 women) recruited in the same manner and using the same inclusion and exclusion criteria as those of Experiment 1 (See **Table 4** for demographic comparisons).

**Table 4:** Comparison of ages, years of education, and scores on the Edinburgh Handedness Inventory (SD) between patients and controls in Experiment 2.

	Patients (*n* = 18)	Controls (*n* = 18)	*t*-test; *P*-value
Age (years)	37.2 (4.8)	35.2 (6.9)	*t* = -1.069; *P* = 0.293
Education (years)	11.5 (2.2)	12.6 (2.1)	*t* = 1.542; *P* = 0.132
Edinburgh score (/20)	18.8 (1.1)	18.5 (1.8)	*t* = -0.800; *P* = 0.429
PANSS positive	13.9 (3.6)	/	/
PANSS negative	17.2 (6.3)	/	/
Illness duration (months)	176.22 (71.4)	/	/

The local ethics committee of Saint-Étienne approved the study (N° IORG0007394), and informed written consent was obtained from all participants.

For the power analysis, see Experiment 1.

#### Apparatus and Materials

Among the 22 everyday objects graspable by one hand used in the previous experiment, six of them were presented in double exemplary (for example two different saucepans, two different bottles of detergent…). In this second experiment, we kept 16 single objects. We presented all objects in two horizontal orientations (compatible with either right- or left-handed grasp) but in only upright orientation. Each object was primed one time by one action sentence congruent with goals of action induced by the use of the object and another time by an incongruent sentence (Appendix 2). We formulated the sentences in relation to the photos of objects used in Experiment 1 by asking 80 students to name the action verb and direct object complement that seemed to them most related to the object in each photo. We used those most often cited (>80%) and subsequently asked the students to form pairs of pictures and sentences that seemed to them most incongruent. The sentences were built to be of almost the same length in French. Overall, in this experiment, 64 pictures were displayed in the middle of a computer screen (with the same characteristics of those in Experiment 1) preceded by sentences written in black 32-point Arial font.

#### Design and Procedure

To ensure that subjects read the sentences presented as primes, we asked them to indicate as fast and accurately as possible if the object pictured (e.g., an iron) was congruent with the action sentence prime (for ironing clothes) or not (for cutting bread). Each participant carried out two blocks of 64 trials with a 3-min break between the blocks. These blocks differed in terms of response mapping (right-hand congruency versus left-hand incongruency and left-hand congruency versus right-hand incongruency) and were counterbalanced between subjects. As in Experiment 1, participants were instructed to keep their right finger on the right response key (L) and their left finger on the left response key (S) during the entire experiment. Response keys were situated 15 cm apart and 20 cm in front of the screen on a standard European (AZERTY) keyboard.

Each experimental trial started with the presentation of an action sentence as a prime for 2000 ms followed by the presentation of a photo of one object, which remained on the screen until an answer was given up to 3000 ms. A brief auditory tone on the computer informed participants of errors. Each participant received eight practice trials using a different set of sentences and photos before each block. Depending on the situation, the response hand could be on the same side as the graspable part of the object (compatible orientation) or on the opposite side (incompatible), and the action sentence prime could be congruent or incongruent with the normal action induced by the given object.

### Results

For all conditions, participants responded within the required time limit of 3000 ms. We evaluated data regarding participants’ errors and response times using ANOVA with group (patients or controls) as the between-subject factor and prime (congruent or incongruent), response (left or right hand), and object orientation (left or right) as within-subject factors.

#### Errors

Errors were rare (*M* = 3.46%; *SE* = 0.4), and the ANOVA showed no significant main effect of either between- or within-subject factors: group [*F*_(1,34)_ = 2.738; *P* = 0.107; η^2^ = 0.07]; prime [*F*_(1,34)_ = 0.386; *P* = 0.539; η^2^ = 0.01]; response [*F*_(1,34)_ = 0.461; *P* = 0.502; η^2^ = 0.01]; and orientation [*F*_(1,34)_ = 2.400; *P* = 0.131; η^2^ = 0.07]. **Table 5** delineates error rates. We found no interaction of factors (all *F*-values were less than 2.400).

**Table 5:** Error rates (SD) based on patient or control group, congruent or incongruent prime, left or right orientation, and left- or right-handed response in Experiment 2.

		Patients		Controls	
Response hand	Object orientation	Primes			
		Congruent	Incongruent	Congruent	Incongruent
Right	Right	4.0 (1.1)	3.7 (1.2)	3.0 (1.1)	2.3 (0.7)
	Left	3.7 (1.3)	5.1 (1.8)	2.0 (0.8)	2.7 (0.9)
Left	Right	4.4 (1.6)	2.7 (0.9)	2.3 (1.0)	2.3 (0.9)
	Left	5.7 (1.7)	4.0 (1.0)	3.7 (1.0)	3.7 (1.2)

#### Response Times

We calculated the mean response time and standard deviation for each subject and excluded RTs above 2 SDs of the individual’s own mean (4.4%).

We found no effect of mapping responses [*F*_(1,34)_ = 1.227; *P* < 0.276; η^2^ = 0.03].

The longer RTs of patients (*M* = 797 ms; *SE* = 35) than controls (*M* = 594 ms, *SE* = 23) reflected a significant main effect of group [*F*_(1,34)_= 22.897; *P* < 0.001; η^2^ = 0.4]. We also found a main effect of prime [*F*_(1,34)_= 28.236; *P* < 0.001; η^2^= 0.5]. Response times were shorter when the prime was congruent with the goal of action induced by the use of the given object (*M* = 672 ms; *SE* = 20) than when the prime was incongruent with it (*M* = 719 ms; *SE* = 23). However, we found no effect of either response [*F*_(1,34)_= 0.663; *P* = 0.421; η^2^= 0.02] or orientation [*F*_(1,34)_= 0.180; *P* = 0.674; η^2^< 0.01]. The only significant interaction was observed between group, prime, response, and orientation [*F*_(1,34)_= 4.254; *P* = 0.047; η^2^= 0.11]. As in Experiment 1, we present separate analyses according to the congruency or incongruency of the prime and the patient or control group (**Table 6**).

**Table 6:** Means (SD) of response times (in ms) according to patient or control group, congruent or incongruent prime, left or right orientation, and left- or right-handed response in Experiment 2.

		Patients		Controls	
Response hand	Object orientation	Primes			
		Congruent	Incongruent	Congruent	Incongruent
Right	Right	757 (38)	837 (36)	552 (18)	621 (33)
	Left	733 (39)	850 (45)	577 (25)	612 (31)
Left	Right	780 (38)	810 (43)	601 (29)	611 (25)
	Left	802 (36)	803 (44)	570 (25)	606 (26)

The 3-way interaction of group × response × orientation was significant when the prime was congruent [*F*_(1,34)_= 8.774; *P* = 0.006; η^2^ = 0.21] but not incongruent [*F*_(1,34)_= 0.340; *P* = 0.563; η^2^ < 0.01]. When the prime was congruent with the goal of action induced by the use of the object, the temporal patterns of response differed significantly according to the participant group.

**FIGURE 3:**
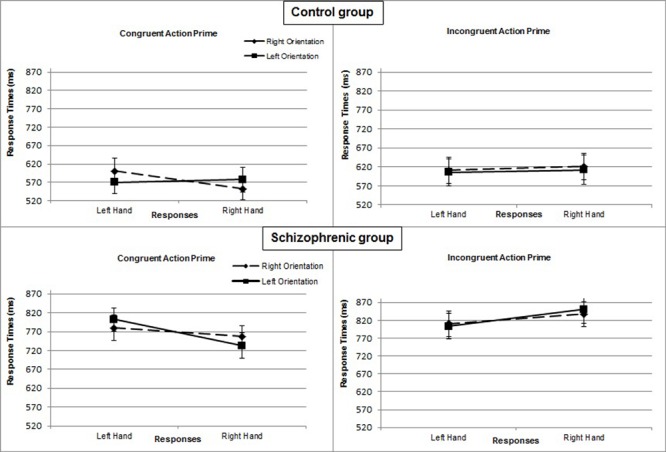
**Mean response times (in ms) for Experiment 2 as a function of action prime (congruent or incongruent), object orientation (left or right), and response hand (left or right) in the control group and in the group with schizophrenia.** Error bars represent standard errors of the mean.

In the control group, the interaction of response × orientation, which measures the effect of compatibility, was not significant [*F*_(1,17)_= 2.890; *P* = 0.107; η^2^= 0.15], but the 3-way interaction of prime × response × orientation [*F*_(1,17)_= 10.359, *P* = 0.005, η^2^= 0.38] was. In the congruent prime condition, the interaction of response × orientation was significant [*F*_(1,17)_= 9.446; *P* = 0.007; η^2^= 0.36]. Right-hand responses were faster when the object was also oriented to the right (*M* = 552 ms; *SE* = 18) rather than to the left (*M* = 577 ms; *SE* = 25) [*F*_(1,17)_= 5.428; *P* = 0.032; η^2^= 0.24]. Similarly, left-hand responses were faster when the object was also oriented to the left (*M* = 570 ms; *SE* = 25) rather than to the right (*M* = 601 ms; *SE* = 29) [*F*_(1,17)_= 5.357; *P* = 0.033; η^2^= 0.24]. By contrast, in the incongruent prime condition, the interaction of response × orientation was not significant [*F*_(1,17)_= 0.061; *P* = 0.808; η^2^< 0.01] (**Figure 3**).

In the patient group, the interaction of response × orientation was not significant [*F*_(1,17)_= 0.666; *P* = 0.426; η^2^= 0.04] and not modified by the prime condition [*F*_(1,17)_= 1.288; *P* = 0.272; η^2^= 0.07], whether congruent [*F*_(1,17)_= 2.505; *P* = 0.132; η^2^= 0.13] or incongruent [*F*_(1,17)_= 0.280; *P* = 0.604; η^2^= 0.02] (**Figure 3**).

### Discussion of Experiment 2

Controls responded more quickly when orientation was compatible between the graspable part of the object and the response hand but only when the prime was a congruent action sentence. An incongruent sentence cued no compatibility effect. These results show that affordance effects can be modulated according to variations in context and particularly according to the goals of action inferred from the experimental setting. Thus, specification of the proper conventional use of an object facilitates the simulation of a particular pattern of motor responses. By contrast, incongruency between the action sentence and perceived object disrupts the affordance effect. These results suggest that if the action implied by a sentence cannot be performed with the object, subjects might not activate the affordances usually provided by the object.

Nevertheless, the subjects with schizophrenia demonstrated no sensorimotor compatibility effect regardless of the congruency of the semantic prime. There was no action potentiation effect even when the sentence and implied action of the object’s use were congruent, and their responses were not faster when the target orientation and response hand were compatible.

However, the relatively low and similar error rates between the two groups of subjects demonstrate the correct understanding of instructions and good involvement to perform the task properly of all participants. These results also highlight that patients had no more difficulty than controls in responding to perceived congruency between the sentence prime and target object, which indicated their understanding of the function of the everyday objects presented to them on the screen. Thus, all participants seemed sensitive to the congruency between the aim of action cued semantically and the perceived object. However, though we observed that patients considered semantic context, the expression of the goal of the action seemed insufficient to create action potentiation during the perception of the objects.

## Discussion

We hypothesized that enriching the contextual environment could influence affordance effects in healthy subjects and facilitate their emergence in patients with schizophrenia. Adapting a paradigm inspired by Tucker and Ellis (1998) to observe the potential modulation of affordance effects, we conducted two experiments in which we introduced a picture of a graspable object using a semantic prime to suggest a sense of ownership of the object (Experiment 1) or goals of action for its use (Experiment 2).

The control group demonstrated the emergence of sensorimotor compatibility effects, but only when the prime was congruent with the perceived object. Indeed, the modulation of the environmental context by conceptual priming influenced the sensorimotor compatibility effects. In Experiment 1, they emerged only when the objects were preceded by the participant’s surname as a reference for ownership of the perceived object, and in Experiment 2, they emerged when action sentences were congruent with goals of action induced by the conventional use of the perceived object. Action potentialities can emerge through simulation mechanisms when the meaning of a stimulus is relevant to the action and when the expected motor response shares components of this action (Girardi et al., 2010).

In our study, context seems to act as a resource to potentiate action when it is congruent with the action implied by the perceived object and with the intention of the subject. The presentation of a congruent prime would enable the preparation of motor action, resulting in the emergence of the affordance effect. Indeed, Vandevoorde (2011) showed that an efficient coupling between the perception of and action associated with an object that is perceived beneficial to the subject will facilitate the reactivation of this kinesthetic image in a similar situation by reinforcement and motor habituation. In this case, the object becomes a “visuomotor opportunity” (Rizzolatti and Sinigaglia, 2008) that is identified based on its motor potentialities. The brain would be able to recognize its environment solely according to these potentialities even in the absence of the superior mobilization of reasoning, so the motor system would then fully participate in identifying and understanding the surrounding world.

Though many studies have focused on the influence of physical context, it seems important to determine the influence of other kinds of context, such as social and functional context, on the activation of affordance (Van Dam et al., 2010; Borghi and Riggio, 2015). In Experiment 1, we observed, we believe for the first time, the potential influence of one’s surname, an example of social and personal data with which we grow from childhood, to simulate an individual’s appropriate actions toward an object when the name is used as a prime for the object’s perception. In Experiment 2, we measured the influence of the input of functional knowledge using action s (“to drink coffee”) to prime the presentation of an object (“a cup”). We wanted to ensure that the participant simulates the expected action: in this example, the most appropriate way to use the cup to drink coffee is to grasp it by its handle. If Van Dam et al.’s (2010, p. 5) showed “*that preparing an action congruent to the typical, functional use of an object, facilitates processing of the word denoting the object,”* then we demonstrated the inverse relationship here.

The results of both our experiments provide further evidence that affordances are both intrinsic to objects and flexible, that they involve the subject and his environment. Even if affordances are initiated automatically, they are then selected to the current task (Borghi and Riggio, 2015). Indeed, we also showed that an incongruent context did not provoke the emergence of affordance effects even when perceived objects were the same. Buccino et al. (2009) tested the modulation of the motor system when an object’s features are violated, such as when the handles of graspable objects are broken, and found no activation of affordance in the absence of pragmatic conditions to perform an action associated with an object. Further studies are needed to detail the mechanisms underlying a total absence of sensorimotor activation or an inhibition of the action potentialities in the case of incongruent context (Anelli et al., 2012; Borghi and Riggio, 2015).

In our subjects with schizophrenia, selected primes did not seem to share sensorimotor features in a relevant way with the current task. Even priming the perception of a visual object using a semantic context to reinforce the sense of property or goals of action of the object did not automatically potentiate the action associated with its use.

In this study, patients were slower than controls in both tasks. Our previous study (Sevos et al., 2013) revealed no such slowdown when we measured the compatibility between the spatial localization of a stimulus and the motor response (Simon task), which seemed to indicate that visuo-spatial integration is automatic in both patients with schizophrenia and healthy subjects. However, in an affordance task, longer response times of patients than controls suggested no automatic binding between perception and action in patients. In that previous work, we interpreted the increased response time as the time to implement controlled processes more costly in attentional resources.

Our current findings again challenge the precept that the mere observation of graspable objects is sufficient to evoke their affordances because objects elicit components of appropriate motor programs associated with object interaction (Borghi and Riggio, 2015). For example, Yu et al. (2014) failed to replicate compatibility effects when participants were not explicitly instructed to imagine picking up pictured objects. However, in both of our experiments, patients with schizophrenia made no more errors than controls, reinforcing the idea that they had functional knowledge of the presented objects-if the object was upright or inverted, if one drinks coffee with a cup or frying pan. Though Garbarini and Adenzato (2004) claim that motor simulation is the only way to develop knowledge of the action possibilities made available by objects, we cannot agree. Indeed, despite the lack of sensorimotor stimulation, our subjects with schizophrenia usually demonstrated the capacity to use everyday life objects in an appropriate way. In this case, simulation could be considered as the default procedure that can occasionally be supplemented or overridden by theoretical considerations, as proposed by Jeannerod and Pacherie (2004).

In pathology, Cattaneo et al. (2007) showed, for example, the inability of autistic children to rely on a motor preparation before executing a movement even if they desired to achieve a requested goal and were fully able to carry out the requested actions. The researchers recorded electromyographic (EMG) activity in children with autism and children with normal development as they executed a gesture (arm flexion toward itself) (Experiment 2). The gesture could lead to two different actions and so involved two different intentions-bringing a piece of food to the mouth (eating action) or putting a piece of paper into a container placed on the shoulder (placing action). Controls demonstrated the increased activity of muscles responsible for the final goal of the action (eating a piece of food) as soon as the action began (reaching for he piece of food). In the children with autism, those muscles became active only during the bringing-to-the-mouth phase. In another experiment (Experiment 1), those authors showed an indirect link between the activity of mirror neurons supposed to support the understanding of the intentions of others and sensorimotor simulation. Using the same procedure, they showed increased activation of jaw muscles as controls observed the eating action but not during the placing action, thus demonstrating the existence of links between motor intention and sensorimotor simulations that activate the muscles involved in the final action. In contrast, the children with autism showed no muscle activation while observing either eating or placing actions. The authors interpret these results as a lack of motor activation underlying action-understanding in children with autism; the children may understand the others’ intentions cognitively (particularly when semantic cues are given by a piece of the object) but not experientially.

Motor facilitation during action observation, which putatively reflects the activity of mirror neurons, could also be reduced in schizophrenia. In their study, Enticott et al.’ (2008) group administered transcranial magnetic stimulation (TMS) while presenting video clips showing the abductor pollicis (APB) of the right hand during different activities (thumb movement, pen grasp, or handwriting) and recorded motor-evoked potentials (MEP) from the right APB muscle of subjects. The significant increase in the amplitude of MEP for these three activities compared with the baseline in controls and the absence of any change in patients with schizophrenia led these authors to conclude that reduced activation of mirror neurons impairs the ability to experience an internal simulation of other’s behavior.

Using our experimental paradigm, associated with physiological measures (TMS or MEP for example), could be relevant to objectify the underlying sensorimotor process in schizophrenia. In the absence of such studies, our behavioral results seem to suggest that our subjects with schizophrenia also have impaired ability to experience an internal simulation of motor action potentialities when they perceived graspable objects, which would indicate that all activities of daily life would require the involvement of higher cognitive processes rather than lower level sensorimotor processes. Patients expressed, for example, knowing how to set a table but needing to think about each step to accomplish the task. Jeannerod (2001), Gallese (2009), and Vandevoorde (2011) agree that it is precisely this sensorimotor simulation that not only enables the subject to anticipate an action but provides as well a “motor thought,” an automatic, almost intuitive knowledge from his surrounding world. This ability to anticipate his actions should allow the subject to act seamlessly in his environment and feel familiar with it and current social situations. At a perceptual level, objects and persons generally appear familiar and intelligible according to our expectations of them from past experience. We postulate that the impairment of sensorimotor simulation could partly explain the loss of this “common sense” of things, sometimes encountered in schizophrenia. If perception appears deprived of its fullness and no longer related to motor actions, but is more like a purely receptive process (Parnas and Handest, 2003), it is not surprising that subjects with schizophrenia can feel a strangeness, that is “*when the meanings of objects in the world (e.g., “What is this chair for?”) and of the actions of others (e.g., “Why is he laughing?”) appear uncanny”* (Stanghellini, 2000, p. 779).

Similarly, Fuchs and Schlimme (2009) claimed that the disintegration of all normally automatic behaviors of everyday life is a major feature of schizophrenia that more broadly reflects a “disembodiment” of the self or of the relation to objects (see also Fuchs, 2005; Stanghellini, 2009; Sass, 2013). Our experimental results seem to converge with clinical observations as well as psychopathological and phenomenological data that demonstrate a dissociation of patients with schizophrenia and their environment.

The study of pathology such as that of schizophrenia, which precisely undermines the notion of coherence, requires a more unified, coherent, and comprehensive approach that takes into account concepts and methods based in embodied cognition (Glenberg et al., 2013).

### Limitations and Implications for Future Studies

This pilot study is limited by our small sample size and the relatively weak positive and negative symptoms of patients that might restrict the generalization of our findings.

Future studies with larger samples of subjects with schizophrenia are warranted to confirm our findings, which suggest the impairment of sensorimotor integration even in patients with milder symptoms.

We explored the emergence of object affordance effects in schizophrenia by varying experimental contexts and found that the use of conceptual priming (making the action more specific or reinforcing the purpose of the action for the patient) seems insufficient to trigger motor simulation when subjects perceive objects used in everyday life. In futures studies, we propose to enhance context by introducing visuomotor or motor priming. Indeed, the literature shows that the state of the motor system can influence the perception of objects (Craighero et al., 2002), but a phase of motor training can also be used to facilitate the effect of sensorimotor compatibility (Borghi et al., 2007).

In addition, the use of brain imaging techniques and electrophysiological measures enhance understanding of the cerebral and physiological mechanisms involved when control subjects perform tasks that involve motor simulation (e.g., Grezes and Decety, 2001, 2002; Buccino et al., 2009). Using such techniques in patients could similarly improve our understanding of these phenomena in schizophrenia.

## Author Contributions

JS and AG designed the study procedures and methods, collected most of the data, conducted all statistical analyses, interpreted the data, wrote the first draft of the manuscript, and edited subsequent versions. DB, JP, and CM proved advice regarding study procedures, methods, and data interpretation and edited the manuscript several times.

## Conflict of Interest Statement

The authors declare that the research was conducted in the absence of any commercial or financial relationships that could be construed as a potential conflict of interest.

The reviewer LT and handling Editor declared their shared affiliation, and the handling Editor states that the process nevertheless met the standards of a fair and objective review.
