# Supplementary material for: Is there any Influence of Variations in Context on Object-Affordance Effects in Schizophrenia? Perception of Property and Goals of Action
Source: Front Psychol. 2016 Oct 5;7:1551. doi: 10.3389/fpsyg.2016.01551 (PMC5050223; doi:10.3389/fpsyg.2016.01551)
Supplement: Supplementary file 1 [file Data_Sheet_1.docx]

**Appendix 1**

The 22 objects used in Experiment 1

Bottle of detergent

Bottle of detergent

Coffee pot

Coffee pot

Cup

Frying pan

Frying pan

Handled dustpan

Iron

Jug

Kettle

Knife

Knife

Mug

Remote control

Saucepan

Saucepan

Small jug

Strainer

Strainer

Watering can

Whisk

**Appendix 2**

The 16 objects used in Experiment 2 with their congruent and incongruent action sentence primes

| **Congruent sentence** | **Object** | **Incongruent sentence** |
| --- | --- | --- |
| *To put the laundry in the washing machine…* | Bottle of detergent | *To cook food…* |
| *To make coffee…* | Coffee pot | *To pour the sauce…* |
| *To drink coffee…* | Cup | *To pick up the crumbs on the floor…* |
| *To cook a steak…* | Frying pan | *To drink coffee…* |
| *To pick up the crumbs on the floor…* | Handled dustpan | *To make coffee…* |
| *To iron clothes…* | Iron | *To serve water…* |
| *To serve water…* | Jug | *To iron clothes…* |
| *To boil water for tea…* | Kettle | *To turn on the TV…* |
| *To cut bread…* | Knife | *To put the laundry in the washing machine…* |
| *To drink hot chocolate…* | Mug | *To cut bread…* |
| *To turn on the TV…* | Remote control | *To water plants…* |
| *To cook food…* | Saucepan | *To boil water for tea…* |
| *To drain pasta…* | Strainer | *To drink hot chocolate…* |
| *To pour the sauce…* | Small jug | *To beat egg whites…* |
| *To water plants…* | Watering can | *To cook a steak…* |
| *To beat egg whites…* | Whisk | *To drain pasta…* |
